# Clinical effects of sodium–glucose cotransporter 2 inhibitors combined with conventional therapy in myocardial infarction: a systematic review and meta-analysis of randomized controlled trials

**DOI:** 10.3389/fcvm.2026.1797628

**Published:** 2026-04-21

**Authors:** Zhipeng Xu, Xinjun Dai, Tianhang Jia, Xi Li, Xiaoyan Zhang, Pengfei Zhang, Huimin Niu, Jie Li

**Affiliations:** 1 Department of Internal Medicine, Lingchuan County Hospital of Traditional Chinese Medicine, Jincheng, China; 2 The Second Affiliated Hospital of Integrated Traditional Chinese and Western Medicine of Hunan University of Chinese Medicine, Changsha, China; 3 Cardiovascular Surgery Department, Xi'an No.1 Hospital, Xi'an, China; 4 Department of Nephrology, Heping Hospital Affiliated to Changzhi Medical College, Changzhi, China; 5 Department of Hematology, Oncology, and Immunology, Affiliated Hospital of Zunyi Medical University, Zunyi, China

**Keywords:** conventional therapy, myocardial infarction, randomized controlled trials, SGLT2i, systematic review and meta-analysis

## Abstract

**Background:**

Sodium–glucose cotransporter 2 inhibitors (SGLT2i) have been shown to improve clinical outcomes in patients with heart failure; however, their efficacy and safety in patients with myocardial infarction, particularly when used in addition to conventional therapy, remain controversial. Therefore, this study aims to evaluate the effects of adding SGLT2i to conventional therapy on clinical outcomes in patients with myocardial infarction through a systematic review and meta-analysis.

**Methods:**

We conducted a systematic review and meta-analysis to compare the effects of conventional therapy with or without SGLT2i on clinical outcomes in patients with myocardial infarction. PubMed, Web of Science, the Cochrane Library, and Embase were systematically searched.

The primary outcome was the incidence of hospitalization for heart failure. Secondary outcomes included all-cause mortality, major adverse cardiovascular events (MACE), left ventricular ejection fraction (LVEF), N-terminal pro-B type natriuretic peptide (NT-proBNP), and low-density lipoprotein cholesterol (LDL-C). Safety outcomes comprised renal dysfunction, hepatic dysfunction, urinary tract infection, and glycemia-related adverse events. All analyses were conducted using a random-effects model. Prespecified subgroup analyses for the primary outcome were performed according to the presence or absence of type 2 diabetes mellitus, timing of SGLT2i initiation, and specific SGLT2i agent.

**Results:**

This meta-analysis included 13 randomized controlled trials involving 22,238 patients with myocardial infarction. Compared with conventional therapy alone, treatment with SGLT-2i significantly reduced the incidence of hospitalization for heart failure (RR = 0.76, 95% CI 0.68–0.84, *p* < 0.00001). For key secondary outcomes, the use of SGLT2i was not associated with all-cause mortality (RR = 0.87, 95% CI 0.75–1.01, *p* = 0.06), but was associated with a significant reduction in the risk of major adverse cardiovascular events (MACE) (RR = 0.84, 95% CI 0.73–0.98, *p* = 0.03). However, no significant difference was observed between the two groups in cardiovascular mortality (RR = 0.87, 95% CI 0.61–1.24, *p* = 0.44). In addition, SGLT2i combined with conventional therapy significantly improved left ventricular ejection fraction (MD = 3.45, 95% CI 0.67–6.24, *p* = 0.02) and significantly reduced NT-proBNP levels (MD = −311.99, 95% CI −666.00–15.23, *p* = 0.04). In terms of safety outcomes, the use of SGLT2i was associated with a reduced risk of renal dysfunction (RR = 0.77, 95% CI 0.66–0.89, *p* = 0.0006) and glycemia-related adverse events (RR = 0.56, 95% CI 0.40–0.80, *p* = 0. 001). No significant increase was observed in the risk of urinary tract infection (RR = 1.73, 95% CI 0.76–3.97, *p* = 0.12; *P* = 0.19) or hepatic dysfunction (RR = 2.46, 95% CI 0.86–6.98, *p* = 0.09; *P* = 0.49). Prespecified subgroup analyses showed that the treatment benefit for the primary outcome was generally consistent irrespective of the presence or absence of type 2 diabetes mellitus or the specific SGLT2i agent used.

**Conclusion:**

In this meta-analysis, the addition of SGLT2i to standard therapy following myocardial infarction was associated with a reduction in hospitalization for heart failure, as well as a lower incidence of renal dysfunction and glycemia-related adverse events. Furthermore, favorable effects of SGLT2i were observed in patients with MI irrespective of the presence or absence of type 2 diabetes mellitus or the specific SGLT2i agent used.

**Systematic Review Registration:**

PROSPERO CRD420251129087.

## Introduction

Myocardial infarction (MI) remains a leading cause of death and disability worldwide and represents the most severe clinical manifestation of coronary artery disease (CAD) (1). According to the 2024 Heart Disease and Stroke Statistics Update issued by the American Heart Association, the overall prevalence of MI among U.S. adults aged ≥20 years is 3.2%, with approximately one MI occurring every 40 s in the United States (2). In a study including 19,781 patients with CAD, the prevalence of MI was reported to be 23.3% (3). Annually, more than 3 million individuals develop ST-segment elevation myocardial infarction (STEMI), affecting over 4 million individuals worldwide (4). In recent years, with continuous refinement of risk stratification systems, widespread implementation of invasive treatment strategies, standardized application of early reperfusion therapy, and broader adoption of secondary prevention measures and individualized therapeutic approaches, the overall prognosis of patients with MI has improved substantially compared with previous decades (5–7). However, owing to the relatively limited introduction of novel therapeutic modalities, the pace of prognostic improvement has gradually slowed. Among patients receiving standard-of-care therapy, the risks of adverse cardiovascular outcomes–including heart failure, stroke, and left ventricular systolic dysfunction–remain considerable, posing a major challenge in contemporary clinical practice (8).

SGLT2i were initially developed as glucose-lowering agents for patients with type 2 diabetes mellitus, primarily by inhibiting glucose reabsorption in the proximal renal tubules, thereby reducing blood glucose levels (9). In recent years, accumulating evidence has shown that SGLT2i exert broad pleiotropic effects in the cardiovascular system, including improvement of myocardial energy metabolism, attenuation of microvascular dysfunction, suppression of inflammatory responses, and preservation of mitochondrial function (10, 11). Based on these mechanisms, SGLT2i have been demonstrated to significantly reduce the composite risk of cardiovascular death or hospitalization for heart failure in patients with heart failure, and these benefits are independent of the presence of diabetes (12, 13).

Although the efficacy of SGLT2i in patients with chronic heart failure has been well established, their clinical role in patients with MI is still being explored. Previous studies have suggested that adding SGLT2i to conventional therapy may reduce the risk of worsening heart failure and hospitalization for heart failure in patients with AMI; however, the results of different randomized controlled trials have been inconsistent (14, 15). The EMPACT-MI trial showed that although empagliflozin added to conventional therapy reduced the total number of hospitalizations for heart failure, it did not reduce the risk of first hospitalization for heart failure or all-cause mortality after AMI (16). Furthermore, whether the specific type of SGLT2i, the presence or absence of concomitant type 2 diabetes mellitus, and the timing of SGLT2i initiation after MI influence its clinical efficacy remains unclear, owing to the lack of systematic evidence synthesis. Therefore, the present study aimed to conduct a systematic review and meta-analysis to evaluate the impact of SGLT2i in addition to standard-of-care therapy, compared with standard-of-care therapy alone, on clinical outcomes in patients with MI.

## Methods

This systematic review and meta-analysis was conducted and reported in accordance with the Preferred Reporting Items for Systematic Reviews and Meta-Analyses (PRISMA) statement and the methodological guidance of the Cochrane Handbook for Systematic Reviews of Interventions (17, 18). The study protocol was registered in PROSPERO (registration number: CRD420251129087), and the study was conducted strictly according to the prespecified protocol, with no major deviations.

### Study selection

Randomized controlled trials published up to July 29, 2025, were included, with the search restricted to studies published in English. Studies were required to meet all of the following inclusion criteria: (1) participants were patients with acute myocardial infarction or a previous history of myocardial infarction; (2) the study compared conventional therapy combined with SGLT2 inhibitors with conventional therapy alone in terms of efficacy and safety; and (3) at least one prespecified efficacy or safety outcome was reported.

Studies that did not report any relevant outcome measures were excluded, as were studies with overlapping populations or duplicate publications. For randomized controlled trials conducted in the general population (including participants with or without type 2 diabetes), studies were included in the analysis only if outcomes for the myocardial infarction subgroup were explicitly reported.

### Search strategy and data extraction

A systematic search was conducted in PubMed, Web of Science, Embase, and the Cochrane Library from database inception to July 29, 2025. The search used predefined keywords including “empagliflozin, dapagliflozin, canagliflozin, ertugliflozin, bexagliflozin”, and “myocardial infarction.” The detailed search strategy for PubMed is provided in Supplementary File.

Two investigators (Z.X. and J.L.) independently screened eligible studies and extracted data. Any disagreements were resolved through discussion involving all coauthors until consensus was reached. Extracted data included author, year, country, study period, interventions, sample size, age, sex, time since myocardial infarction, diabetes status, type of SGLT2i, estimated glomerular filtration rate (eGFR), study name, duration of follow-up, and outcome measures.

### Primary and secondary outcomes

The primary outcome of this study was the total rate of hospitalization for heart failure (HF) among patients with myocardial infarction (myocardial infarction, MI). Secondary outcomes included all-cause mortality (ACM), major adverse cardiovascular events (MACE) (Table 1), left ventricular ejection fraction, N-terminal pro–B-type natriuretic peptide (NT-proBNP), and low-density lipoprotein cholesterol (LDL-C). Adverse events were systematically assessed, including hepatic dysfunction (defined as any reported liver injury), renal dysfunction (defined as any reported renal impairment or adverse events leading to drug discontinuation), urinary tract infection, and glycemia-related adverse events (including hypoglycemia and/or ketoacidosis of any severity). Major adverse cardiovascular events (MACE) were defined as a composite endpoint of cardiovascular death, myocardial infarction, or stroke. All outcomes were analyzed in accordance with the definitions provided in the original studies.

**Table 1:** The detailed definitions of each endpoint.

Outcome	Definition
Heart Failure Hospitalization	Hospital admission due to any heart failure related symptom, lab/imaging findings or physical signs. The hospitalization required to be reported by an investigator. Heart failure hospitalizations were also considered as a worsening in heart failure episodes
All-Cause Mortality	All cause mortality is defined as any cause of death in a targeted population over a period of time
Major Adverse Cardiovascular Event (MACE)	MACE was defined as cardiovascular death (CV death), myocardial infarction (MI) or all cause stroke
Cardiovascular (CV) Death	CV deaths include those resulting from a myocardial infarction (MI), sudden cardiac death, heart failure (HF), stroke, cardiovascular procedures, cardiovascular hemorrhage, and other CV causes
Hepatic dysfunction	Any degree of hepatic injury
glycemia-related adverse events	Any degree of hypoglycemia and diabetic ketoacidosis
Renal dysfunction	Any renal-related adverse event or adverse event leading to discontinuation of treatment

Patients were stratified according to the timing of SGLT2 inhibitor initiation (within 8 weeks vs. beyond 8 weeks after myocardial infarction). This time threshold was defined based on prior evidence indicating that ventricular remodeling is most pronounced within approximately 8 weeks following MI (19). Therefore, this stratification may help to evaluate the impact of the timing of SGLT2 inhibitor initiation on clinical benefit.

The left ventricular ejection fraction (LVEF), N-terminal pro-B-type natriuretic peptide (NT-proBNP), and low-density lipoprotein cholesterol (LDL-C) have different reference ranges in the studies included. In this meta-analysis, we did not standardize these definitions but instead processed the data according to the measurement standards used in each individual study.

### Quality assessment

Six authors (Z.X.; X.D.; Y.Z.; P.Z.; X.Z.; H.N.) were randomly assigned into three pairs and independently assessed the risk of bias of each study using the Revised Cochrane risk-of-bias tool for randomized trials (RoB 2), as recommended by the Cochrane Handbook for Systematic Reviews of Interventions (20, 21). Any disagreements were resolved through discussion to reach consensus; if necessary, other authors (X.L.; J.L.) were consulted to resolve discrepancies.

### Statistical analysis

The outcomes included both dichotomous and continuous variables. When outcomes were measured on the same scale, effect sizes were expressed as mean difference (MD) or risk ratio (RR). Continuous variables were analyzed using the mean difference (MD) with 95% confidence intervals (CIs), whereas categorical variables were analyzed using risk ratios (RRs) with 95% CIs. Given the potential clinical and methodological heterogeneity across the included trials (e.g., differences in study populations, duration of follow-up, and endpoint definitions), a random-effects model was systematically applied for all analyses.

Heterogeneity was assessed for each outcome using the I^2^ statistic and the Cochrane Q test with Review Manager software (version 5.4.1). An I^2^ value of 0%–25% with *P* > 0.10 indicated no heterogeneity; 25%–50% with *P* > 0.10 indicated moderate heterogeneity; 50%–75% or *P* < 0.10 indicated substantial heterogeneity; and 75%–100% or *P* < 0.10 indicated considerable heterogeneity (18, 20).

Publication bias was assessed using Egger's test. When an outcome included ten or more studies, a funnel plot was generated to visually inspect asymmetry.

The quality of evidence for each outcome was evaluated using the Grading of Recommendations Assessment, Development and Evaluation (GRADE) approach. Based on the overall performance across the 5 GRADE domains, the certainty of evidence was classified as high, moderate, low, or very low (22). All statistical analyses were performed using Review Manager (version 5.4.1) and Stata (version 17).

## Results

### Literature search and baseline characteristics

**Figure 1:**
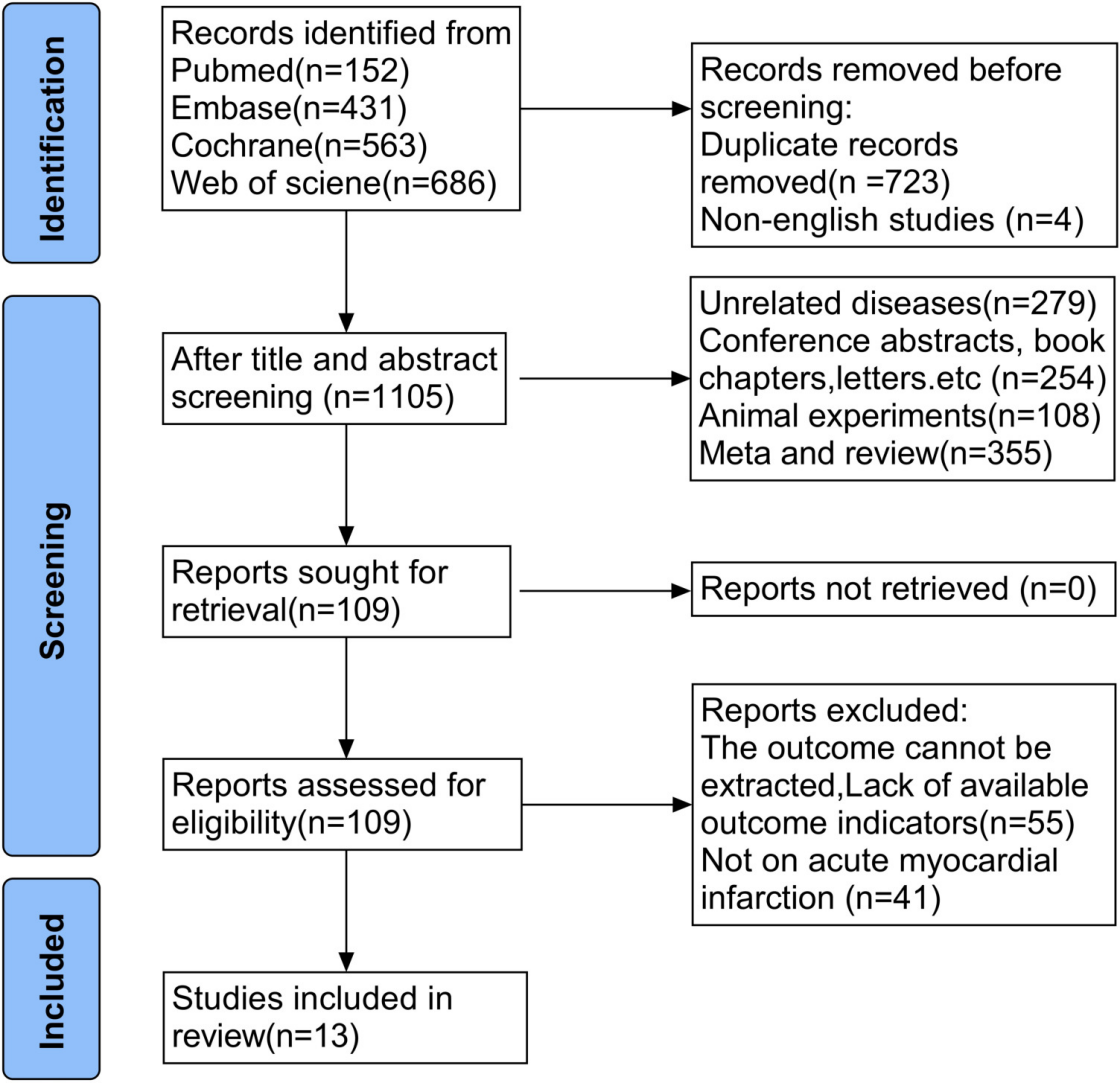
The literature search and study selection process.

The literature search and study selection process are shown in Figure 1. A total of 1,832 records were identified through systematic searches of PubMed, Web of Science, the Cochrane Library, and Embase. After removing duplicate records and non-English publications, 1,105 studies remained for title and abstract screening. Following further screening, 109 studies were assessed in full text, of which 55 were excluded for not meeting the inclusion criteria and 41 were excluded because the primary disease studied was not acute myocardial infarction. Ultimately, 13 randomized controlled trials, including a total of 22,238 patients with myocardial infarction, were included in the final meta-analysis (15, 16, 19, 23–33). Among the included studies, 8 evaluated empagliflozin, 5 evaluated dapagliflozin (23). The baseline characteristics and key design features of the included studies are summarized in Table 2, including study region, sample size, duration of follow-up, patient demographic characteristics, timing of SGLT2i initiation, presence or absence of diabetes, and details of the intervention.

**Table 2:** The baseline characteristics and key design features of the included studies.

	Year	Trial	Follow time	Type of SGLT2i	Sample size	median Age (Years)		Male		DM II		Time of MI	eGFR	
						SGLT2i	Non SGLT2i	SGLT2i	Non SGLT2i	SGLT2i	Non SGLT2i		SGLT2i	Non SGLT2i
1	2020	EMBODY	5.5months	Empagliflozin 10 mg	96	63.9	64.6	38 (82.6)	39 (78.0)	100%	100%	<2 weeks	64.60 (14.95)	66.14 (15.72)
2	2024	EMPACT-MI	17.9 months	Empagliflozin 10mg	6,522	63.6	63.6	2,448	2,449	1,035 (31.7)	1,046 (32.1)	<2 weeks	77.5 (62.2–91.0)	78.0 (61.7–91.4)
3	2024	DELIVER + DAPA-HF	27.6 months	Dapagliflozin 10 mg	3,731	68.7		2,825 (75.7)		1,835 (49.2)		≤3 months	62.9 ± 18.8	
4	2019	DECLARE-TIMI 58	50.4 months	Dapagliflozin 10 mg	3,584	62		2739		100%		>8 weeks	88 (73–97)	
5	2025	EMPRESS-MI	24 weeks	Empagliflozin 10 mg	105	63.4	62.6	44 (86.3)	46 (86.8)	4 (7.8)	5 (9.4)	12 h to 14 days	78.3 (20.3)	79.3 (20.2)
6	2022	EMMY	6.5 months	Empagliflozin 10 mg	476	57	57	195 (82)	197 (82)	30 (13)	30 (13)	≤72 h	92 (78–101)	92 (78–101)
7	2022	Adel	6 months	Empagliflozin 10 mg	93	55	55	27 (60.0)	29 (60.4)	100%	100%	After PCI	N/A	N/A
8	2024	EMI-STEMI	40 days	Empagliflozin 10 mg	101	59.2	61.6	39 (78.0)	40 (78.4)	0%	0%	primary PCI	78.4 (19.8)	78.4 (19.8)
9	2023	DACAMI	2.8months	Dapagliflozin 10 mg	100	55.24	56.7	42 (84.0%)	41 (82.0%)	0%	0%	≤72 h after pPCI	82.61 ± 14.31	85.49 ± 13.49
10	2023	DAPA MI	11.6 months	Dapagliflozin 10 mg	4,017	63	62.8	1,631 (80.8)	1,579 (79.0)	0%	0%	7–10 days	83.5 ± 17.12	83.4 ± 16.91
11	2019	EMPA-REG OUTCOME^a^	37.2months	Empagliflozin 10 mg or 25 mg	7,020	63.1	63.2	3,336 (71.2)	1,680 (72.0)	100%	100%	–	N/A	N/A
12	2025	Le Zhou	24 weeks	Dapagliflozin 10 mg	98	68.05	68.05	28 (58.33%)	30 (60%)	25	30	–	N/A	N/A
13	2022	SOCOGAMI	7 months	Empagliflozin 25 mg	42	67	68	16	16	5	10%	<6 months	68 ± 13	73 ± 14

^a^ Data reported from entire study population, not only myocardial infarction patients. The number of individuals who have experienced myocardial infarction is 3,273.

SGLT-2i, sodium-glucose cotransporter 2 inhibitors; DM II, type 2 diabetic patient; MI, myocardial infarction; eGFR, estimated glomerularfiltrationrate; PCI, percutaneous coronary intervention.

### Quality assessment

**Figure 2:**
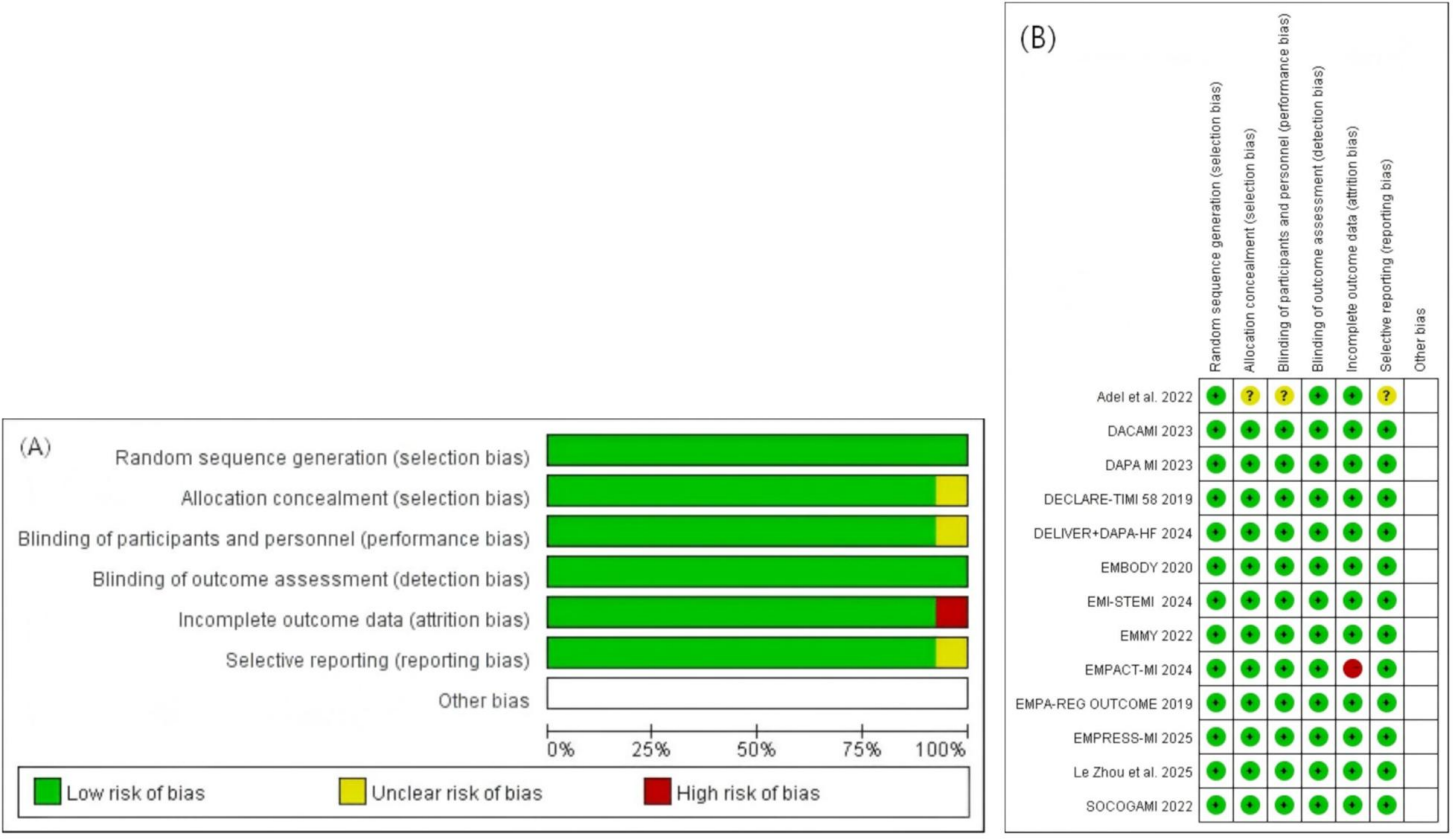
Quality assessment. **(A)** Risk of bias graph; **(B)** risk of bias summary.

As shown in Figure 2, all 13 included randomized controlled trials employed random sequence generation. 12 studies were well designed, with no apparent methodological flaws, and all domains were judged to be at low risk of bias. However, the EMPACT-MI 2024 study exhibited issues related to missing outcome data (attrition bias); therefore, this domain was assessed as being at high risk of bias.

### Pooled analysis of all studies

#### Primary outcomes

**Figure 3:**
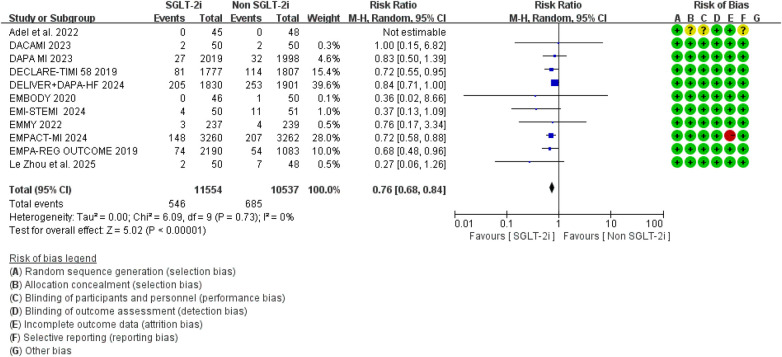
The pooled results of hospitalization for heart failure (HF). SGLT-2i, sodium-glucose cotransporter 2 inhibitors; CI, confidence interval.

**Figure 4:**
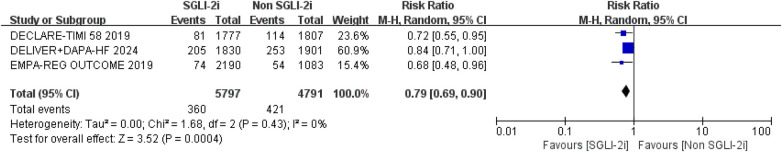
The pooled results of SGLT-2i were initiated within 8 weeks after MI. SGLT-2i, sodium-glucose cotransporter 2 inhibitors; CI, confidence interval.

**Figure 5:**
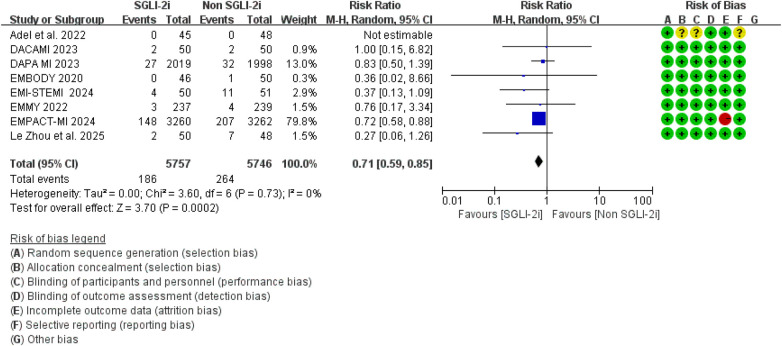
The pooled results of SGLT-2i were initiated beyond 8 weeks after MI. SGLT-2i, sodium-glucose cotransporter 2 inhibitors; CI, confidence interval.

The primary outcome was hospitalization for heart failure (HF), and the pooled results are shown in Figure 3. A total of 12 randomized controlled trials reported HF-related hospitalization events. Compared with conventional therapy alone, conventional therapy combined with SGLT2 inhibitors significantly reduced the risk of HF-related hospitalization (RR = 0.76, 95% CI 0.68–0.84, *p* < 0.00001). No significant heterogeneity was observed among the included studies (*I*^2^ = 0.0%, *P* = 0.81). Separate analyses of studies in which SGLT2i were initiated within 8 weeks and beyond 8 weeks after MI showed effect estimates that were broadly comparable to those of the overall pooled analysis, with no evidence of substantial heterogeneity (Figures 4, 5). Notably, the three studies evaluating initiation beyond 8 weeks were not specifically designed for patients with MI.

**Figure 6:**
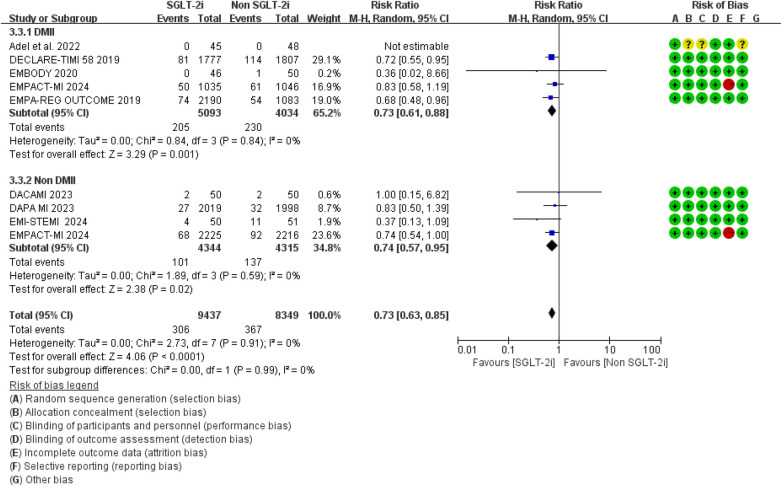
The pooled results of subgroup analyses based on whether DMII was combined or not. SGLT-2i, sodium-glucose cotransporter 2 inhibitors; CI, confidence interval.

The addition of SGLT2 inhibitors to conventional therapy reduced the risk of hospitalization for heart failure irrespective of type 2 diabetes mellitus status (Figure 6). The *p* value of the interaction is greater than 0.05, indicating that the hospitalization rate for heart failure is not affected by whether patients with myocardial infarction have diabetes.

**Figure 7:**
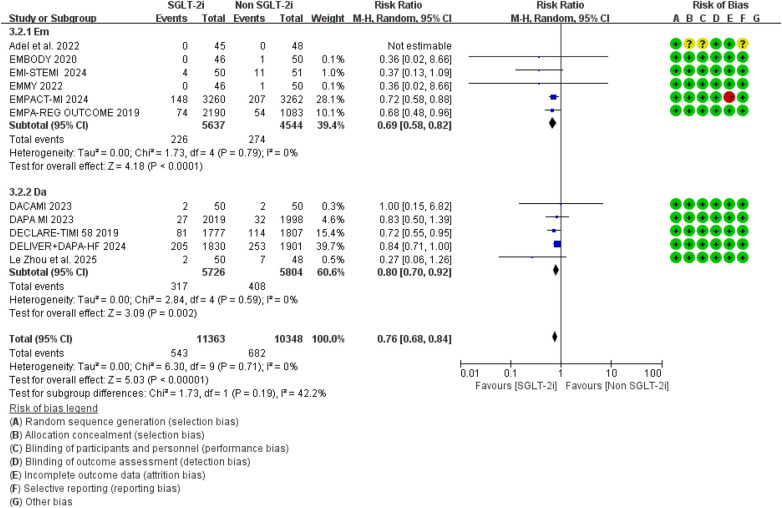
The pooled results of subgroup analyses based on the types of SGLT2i. SGLT-2i, sodium-glucose cotransporter 2 inhibitors; CI, confidence interval.

In addition, subgroup analyses according to the specific SGLT2 inhibitor suggested that, compared with conventional therapy alone, the addition of empagliflozin or dapagliflozin to standard therapy was associated with a trend toward a reduced risk of hospitalization for heart failure (Figure 7). No significant interaction was observed between SGLT2i type and treatment effect (*P* for interaction >0.05) (Table 3), suggesting that the magnitude of benefit may not differ significantly according to the specific agent used.

**Table 3:** Subgroup analysis of the hospitalization rate related to HF.

Subgroup	Number of included studies	Pooled effects			*p* value for interaction	Heterogeneity	
		RR	95% CI	*p* value		*I*^2^,%	*p* value
T2DM	5	0.73	[0.61, 0.88]	**0**.**001**	0.803	0	0.84
Non T2DM	4	0.74	[0.57, 0.95]	**0**.**02**		0	0.59
Em	6	0.69	[0.58, 0.82]	**<0**.**0001**	0.285	0	0.79
Da	5	0.80	[0.70, 0.92]	**0**.**002**		0	0.59

Bold values indicate statistically significant values at *p* < 0.05.

SGLT-2i, sodium–glucose cotransporter 2 inhibitors; RR, risks ratio; CI, confidence interval; T2DM, type 2 diabetes mellitu; MI, myocardial infarction; En, empagliflozin; Da, dapagliflozin.

### Secondary outcomes

**Figure 8:**
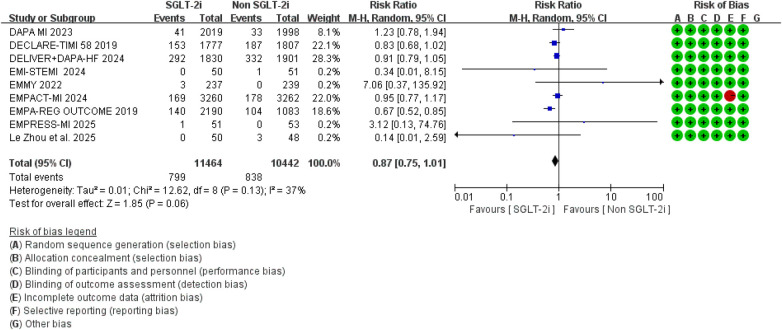
The pooled results of all-cause mortality. SGLT-2i, sodium-glucose cotransporter 2 inhibitors; CI, confidence interval.

**Figure 9:**
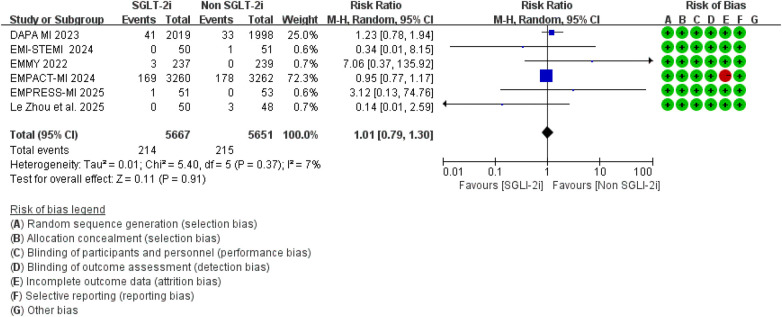
The pooled results of studies specifically designed for patients with MI. SGLT-2i, sodium-glucose cotransporter 2 inhibitors; CI, confidence interval.

A total of 9 studies reported data on all-cause mortality. Pooled analysis showed that, compared with conventional therapy alone, the addition of SGLT2 inhibitors to standard therapy was not significantly associated with all-cause mortality (RR = 0.87, 95% CI 0.75–1.01, *p* = 0.06), with moderate between-study heterogeneity (*I*^2^ = 37%, *P* for heterogeneity = 0.13) (Figure 8). Analyses restricted to studies specifically designed for patients with MI showed broadly comparable findings (Figure 9).

**Figure 10:**
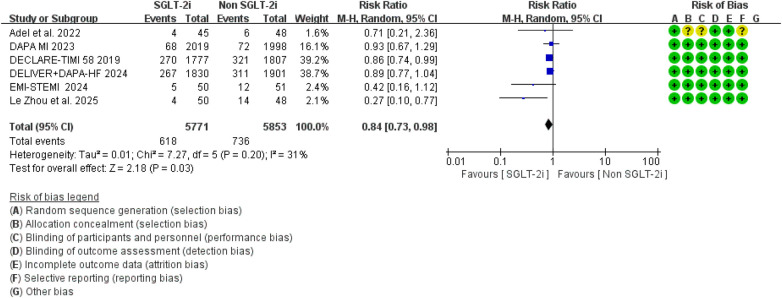
The pooled results of major adverse cardiovascular events (MACE). SGLT-2i, sodium-glucose cotransporter 2 inhibitors; CI, confidence interval.

Regarding major adverse cardiovascular events (MACE), seven randomized controlled trials reported relevant outcomes. The pooled analysis showed that, compared with conventional therapy alone, conventional therapy combined with SGLT2 inhibitors was associated with a significantly lower risk of MACE (RR = 0.84, 95% CI 0.73–0.98, *p* = 0.03), with moderate heterogeneity observed among studies (*I*^2^ = 31%, *P* = 0.20) (Figure 10).

**Figure 11:**
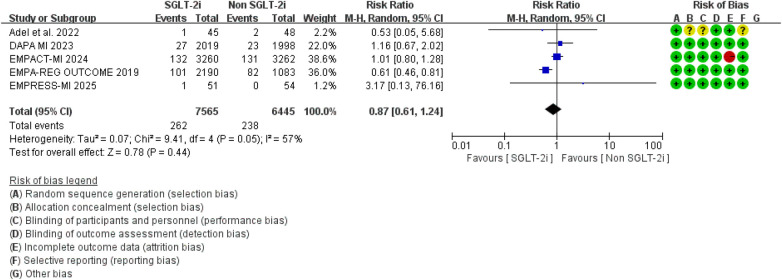
The pooled results of cardiovascular mortality. SGLT-2i, sodium-glucose cotransporter 2 inhibitors; CI, confidence interval.

**Figure 12:**
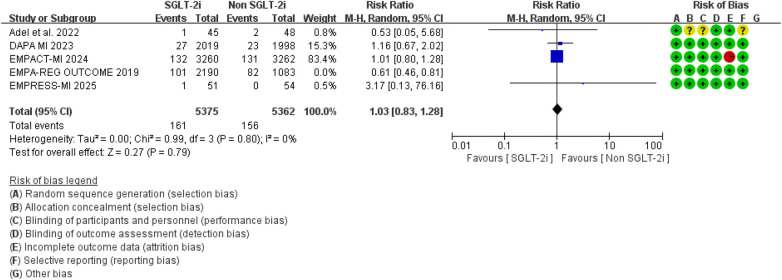
Sensitivity analysis of the cardiovascular mortality outcomes. SGLT-2i, sodium-glucose cotransporter 2 inhibitors; CI, confidence interval.

However, no significant difference was observed between the two groups regarding cardiovascular mortality outcomes (RR = 0.87, 95% CI 0.61–1.24, *p* = 0.44), and there was considerable heterogeneity between studies (*I*^2^ = 57%, *P* = 0.05) (Figure 11). Sensitivity analysis indicated that the “EMPA-REG OUTCOME” study might contribute to the observed heterogeneity. After excluding this study, the overall result remained unchanged, but the heterogeneity was notably reduced (Figure 12).

**Figure 13:**
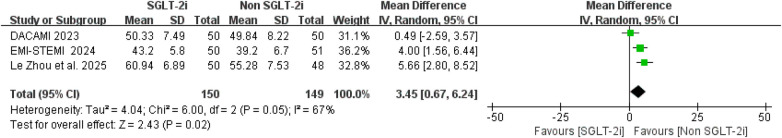
The pooled results of left ventricular ejection fraction. SGLT-2i, sodium-glucose cotransporter 2 inhibitors; CI, confidence interval.

Regarding cardiac function and biomarkers, the use of SGLT2 inhibitors was associated with an improvement in left ventricular ejection fraction (MD = 3.45, 95% CI 0.67–6.24, *p* = 0.02), although considerable heterogeneity was observed between studies (*I*^2^ = 67%, *P* = 0.05) (Figure 13).

**Figure 14:**
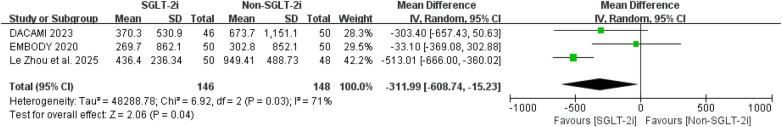
The pooled results of N-terminal pro-B type natriuretic peptide (NT-proBNP). SGLT-2i, sodium-glucose cotransporter 2 inhibitors; CI, confidence interval.

Additionally, compared to conventional therapy alone, the combination of SGLT2 inhibitors significantly reduced NT-proBNP levels (MD = −311.99, 95% CI −608.74 to −15.23, *p* = 0.04), although considerable heterogeneity was observed between studies (*I*^2^ = 71%, *P* = 0.03) (Figure 14).

**Figure 15:**
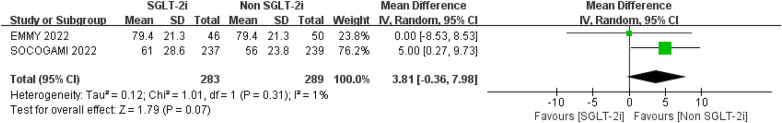
The pooled results of low-density lipoprotein cholesterol (LDL-C). SGLT-2i, sodium-glucose cotransporter 2 inhibitors; CI, confidence interval.

No significant difference was observed between the two groups regarding LDL-C levels (MD = 3.81, 95% CI −0.36 to 7.98, *p* = 0.07), and no significant heterogeneity was detected between studies (*I*^2^ = 1%, *P* = 0.31) (Figure 15).

### Adverse drug reactions

**Figure 16:**
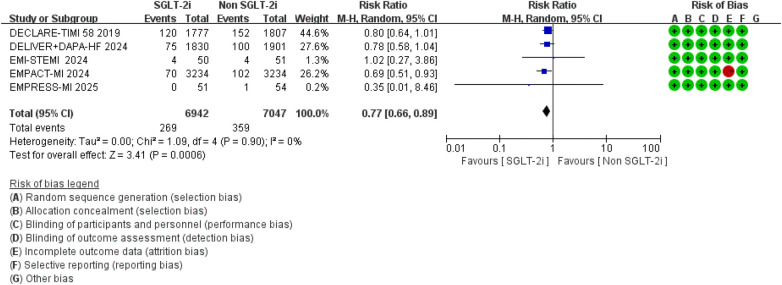
The pooled results of renal impairment. SGLT-2i, sodium-glucose cotransporter 2 inhibitors; CI, confidence interval.

Regarding adverse events, the meta-analysis results showed that, compared to conventional therapy alone, the combination of conventional therapy and SGLT2 inhibitors was associated with a reduced risk of renal impairment (RR = 0.77, 95% CI 0.66–0.89, *p* = 0.0006), with no significant heterogeneity observed between studies (*I*^2^ = 0.0%, *P* = 0.90) (Figure 16).

**Figure 17:**
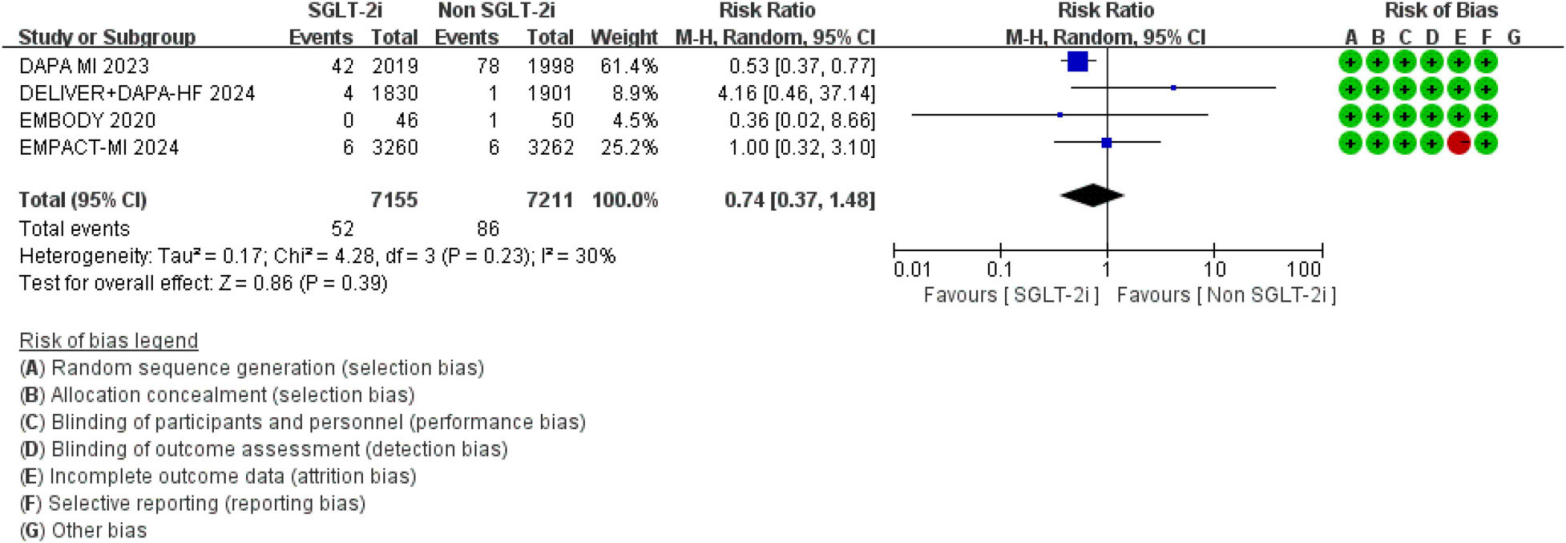
The pooled results of glucose-related adverse events. SGLT-2i, sodium-glucose cotransporter 2 inhibitors; CI, confidence interval.

Similarly, the combination of SGLT2 inhibitors and conventional therapy was associated with a 44% reduction in the occurrence of glucose-related adverse events (RR = 0.56, 95% CI 0.40–0.80, *p* = 0.01), with no significant heterogeneity observed between studies (*I*^2^ = 0%, *P* = 0.56) (Figure 17).

**Figure 18:**
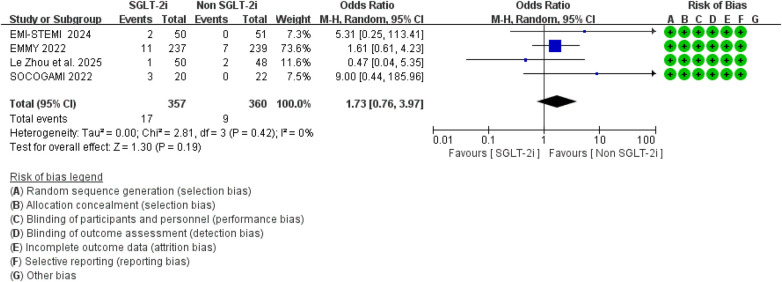
The pooled results of urinary tract infections. SGLT-2i, sodium-glucose cotransporter 2 inhibitors; CI, confidence interval.

**Figure 19:**
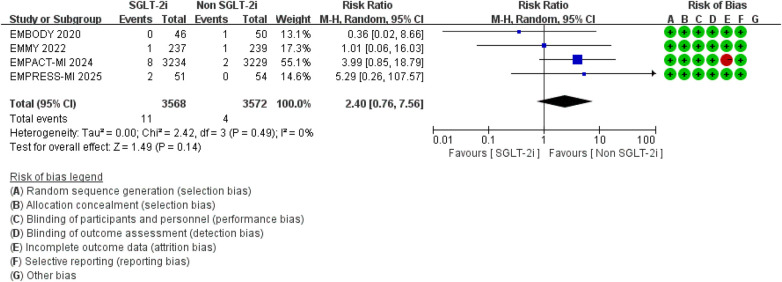
The pooled results of liver function impairment. SGLT-2i, sodium-glucose cotransporter 2 inhibitors; CI, confidence interval.

Additionally, compared to conventional therapy alone, the addition of SGLT2 inhibitors did not significantly increase the risk of urinary tract infections (RR = 1.73, 95% CI 0.76–3.97, *p* = 0.19; *I*^2^ = 0%, *P* = 0.42) or liver function impairment (RR = 2.40, 95% CI 0.26–7.56, *p* = 0.14; *I*^2^ = 0%, *P* = 0.49) (Figures 18, 19).

### Sensitivity analysis

**Figure 20:**
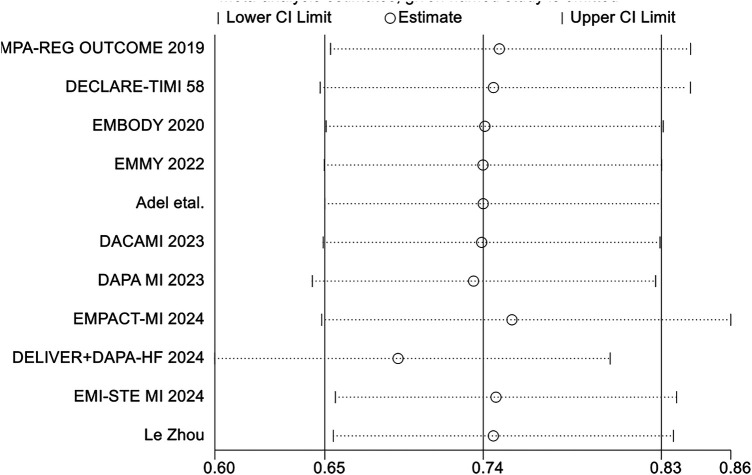
Sensitivity analyses for heart failure-related hospitalizations (Figure 20).

**Figure 21:**
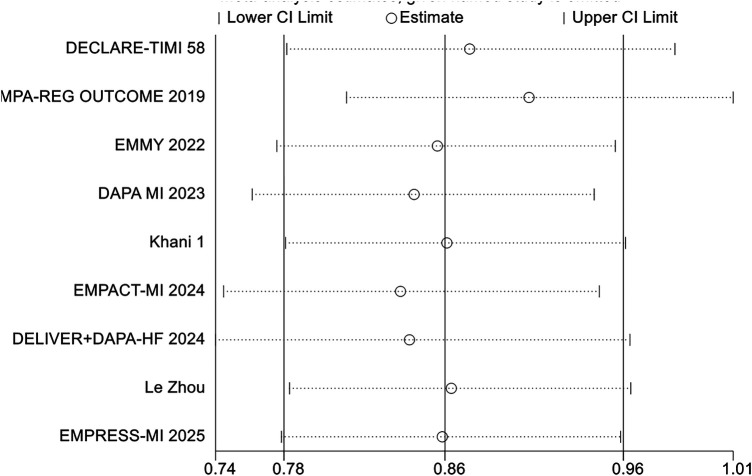
Sensitivity analyses for all-cause mortality (Figure 21).

**Figure 22:**
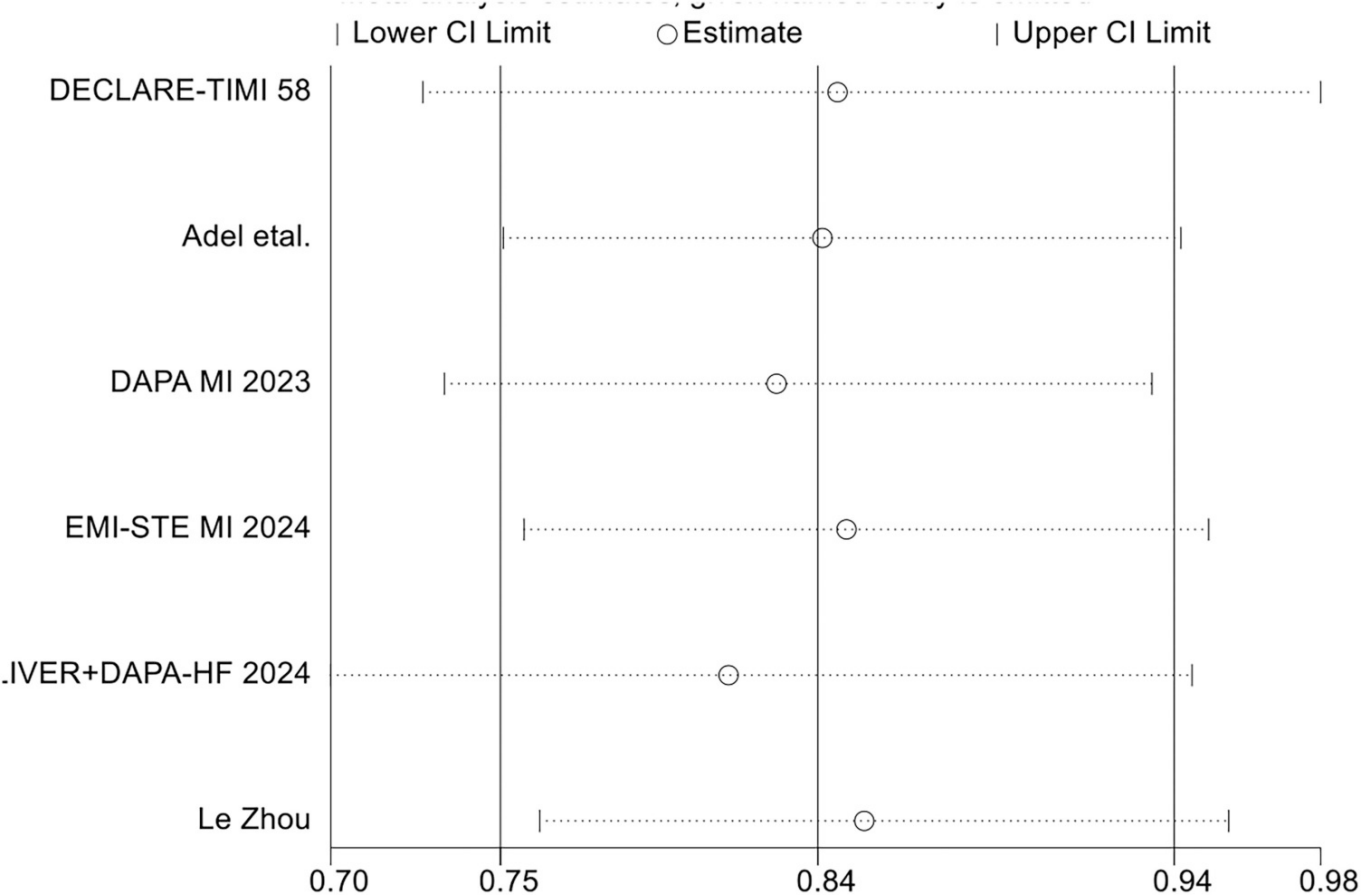
Sensitivity analyses for major adverse cardiovascular events (Figure 22).

**Figure 23:**
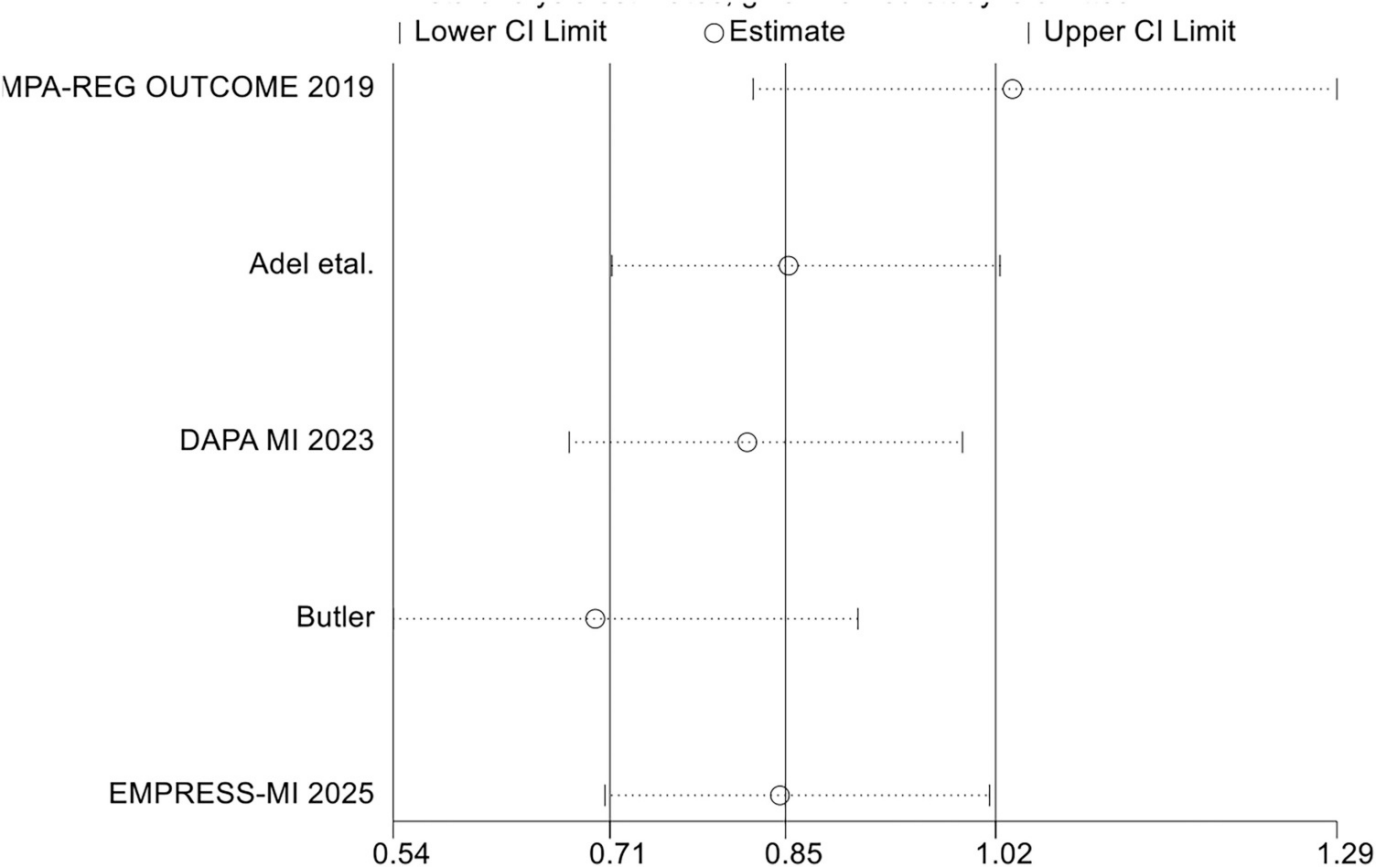
Sensitivity analyses for cardiovascular mortality (Figure 23).

Sensitivity analyses for heart failure-related hospitalizations (Figure 20), all-cause mortality (Figure 21), major adverse cardiovascular events (Figure 22), and cardiovascular mortality (Figure 23) indicated that after sequentially excluding individual studies, the overall pooled effect remained consistent. For heart failure-related hospitalizations and all-cause mortality, the results of separate analyses for “studies designed for MI” and “studies not designed for MI” were consistent with those of the combined analysis. However, due to the limited number of included studies, although the results partially support the stability of the effect estimates, these findings should be interpreted with caution.

### Publication bias

**Figure 24:**
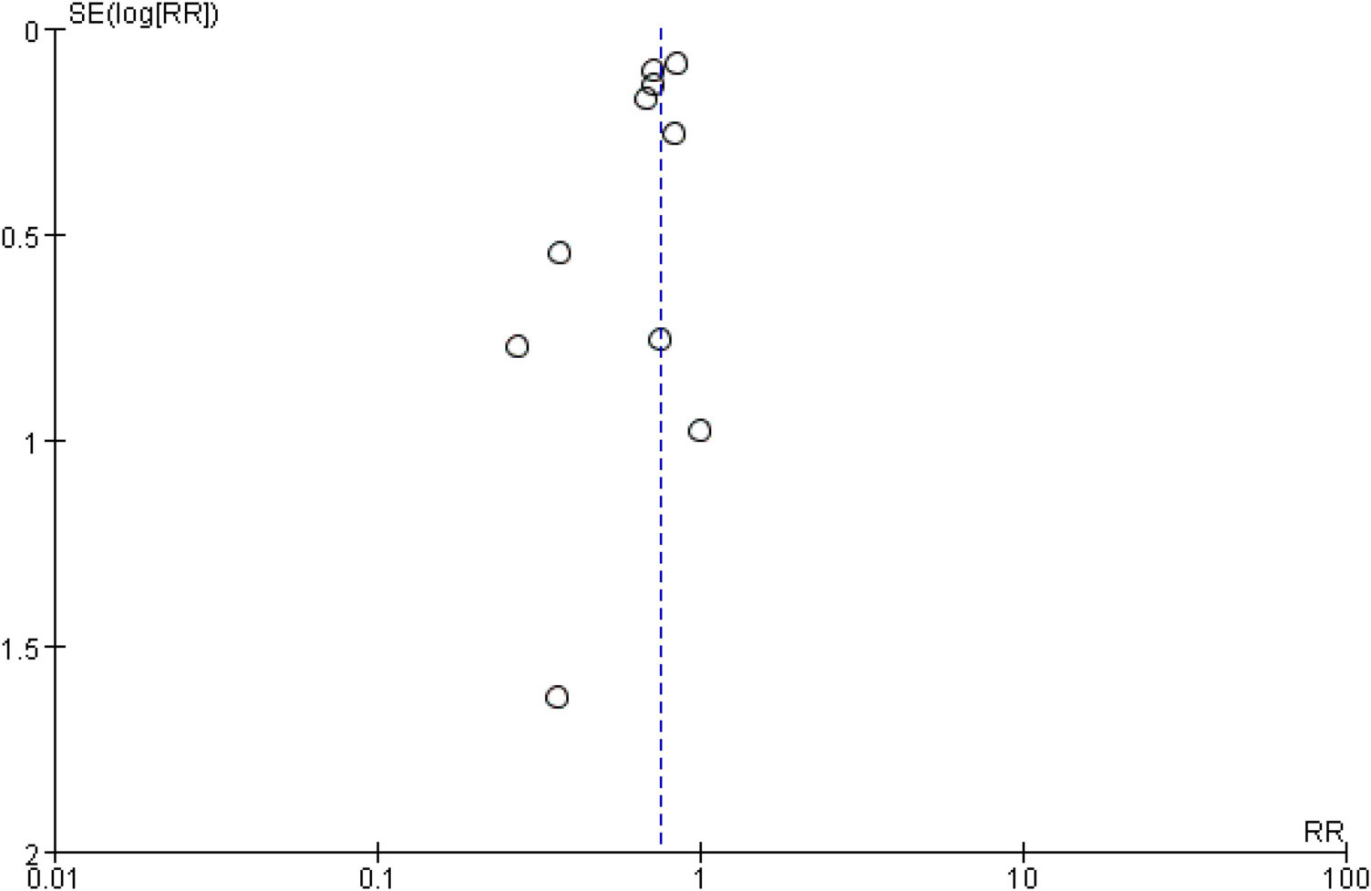
The funnel plots for total hospitalization rates for heart failure (HF).

**Figure 25:**
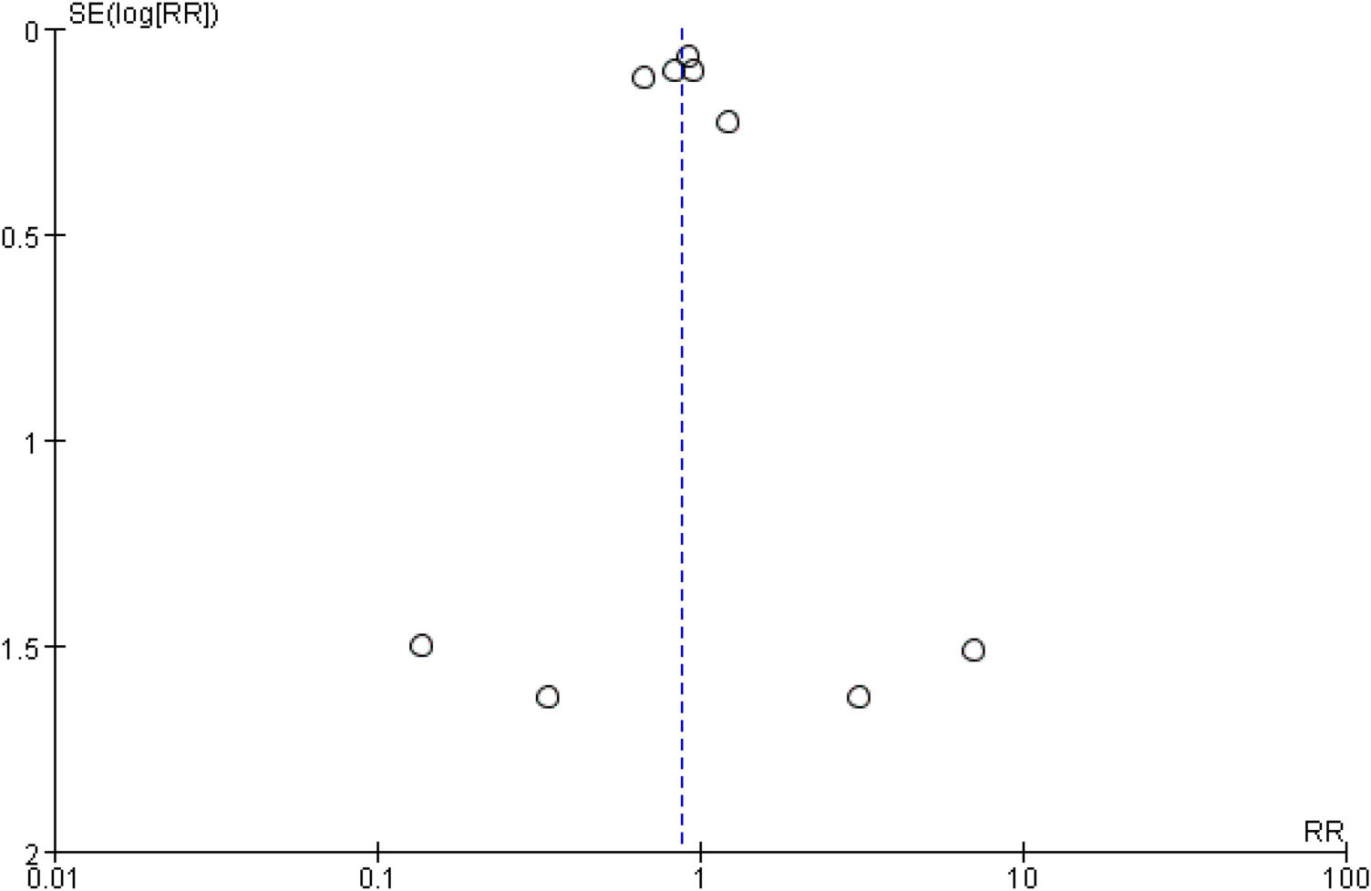
The funnel plots for total hospitalization rates for all-cause mortality (ACM).

Funnel plots and Egger's test were employed to assess publication bias. The funnel plots for total hospitalization rates for heart failure (HF) and all-cause mortality (ACM), based on 10 or more studies, showed symmetry (Figures 24, 25). The results of Egger's test indicated that no significant publication bias was detected for heart failure total hospitalization rates (*p* = 0.098) and all-cause mortality (*p* = 0.736).

### GRADE rating

Based on the GRADE (Grading of Recommendations Assessment, Development, and Evaluation) approach, a systematic evaluation of the certainty of evidence for all included binary and continuous outcomes in patients with myocardial infarction was conducted. The results showed that the certainty of evidence for glucose-related adverse events was low; for liver function impairment, NT-proBNP, LVEF, and cardiovascular death, the certainty of evidence was moderate; and for urinary tract infections, renal impairment, major cardiovascular events, all-cause mortality, and total hospitalization rate for heart failure, the certainty of evidence was rated as high. The detailed GRADE ratings are shown in Table 4.

**Table 4:** GRADE rating of each outcome.

No. of studies	Outcomes	Metrics	Estimate	95%CI	I2; *P* value	Risk of bias	Inconsistency	Indirectness	Imprecision	Publication bias	Plausible confounding	Magnitude of effect	Dose-response gradient	GRADE
12	Total hospitalization rate for HF	RR	0.75	0.68, 0.84	0.0%; *P* = 0.81	No serious risk	No serious	No serious	No serious	Undetected	Would not reduce effect	NO	NO	High
10	All-Cause Mortality	RR	0.88	0.81, 0.96	31.3%; *P* = 0.16	No serious risk	No serious	No serious	No serious	Undetected	Would not reduce effect	NO	NO	High
7	Major cardiovascular events	RR	0.87	0.79, 0.96	29.1%; *P* = 0.21	No serious risk	No serious	No serious	No serious	Undetected	Would not reduce effect	NO	NO	High
4	Poor blood glucose control	RR	0.60	0.43, 0.85	30.0%; *P* = 0.23	No serious risk	No Serious	No serious	No serious	Undetected	Would not reduce effect	NO	NO	Low
4	Liver function impairment	RR	2.46	0.86, 6.98	90.0%;*P* = 0.49	No serious risk	Serious inconsistency	No serious	No serious	Undetected	Would not reduce effect	NO	NO	Moderate
3	NT-proBNP	SMD	−0.46	−0.76, −0.28	96.0%;*P* < 0.00001	No serious risk	Serious inconsistency	No serious	No serious	NA	Would not reduce effect	NO	NO	Moderate
3	LVEF	SMD	0.48	0.25, 0.72	71.7%;*P* = 0.03	No serious risk	Serious inconsistency	No serious	No serious	Undetected	Would not reduce effect	NO	NO	Moderate
5	Renal impairment	RR	0.76	0.65, 0.89	0.0%; *P* = 0.89	No serious risk	No serious	No serious	No serious	Undetected	Would not reduce effect	NO	NO	High
6	CV death	RR	0.94	0.68, 1.31	57.8%; *P* = 0.04	No serious risk	Serious inconsistency	No serious	No serious	Undetected	Would not reduce effect	NO	NO	Moderate
4	Urinary tract infections	RR	1.82	0.86, 3.88	0.0%; *P* = 0.44	No serious risk	No serious	No serious	No serious	Undetected	Would not reduce effect	NO	NO	High
2	LDL-C	SMD	0.16	−0.01, 0.32	0.0%; *P* = 0.40	No serious risk	No serious	No serious	serious	Undetected	Would not reduce effect	NO	NO	Moderate

RR, relative risk; 95%CI, 95% confidence interval; SMD, standard mean difference; GRADE, grade of recommendations assessment development and evaluation; HF, heart failure; LVEF, left ventricular ejection fractions; CV death, cardiovascular death; LDL-C, low-density lipoprotein cholesterol; NT-proBNP, N-terminal pro-hormone of brain natriuretic peptide; NA, not applicable.

## Discussion

Based on the growing body of evidence from evidence-based medicine, SGLT2 inhibitors have been included in the 2025 Global Implementation Guidelines for Heart Failure by the iCARDIO Alliance, becoming a cornerstone in the treatment of both heart failure with reduced ejection fraction (HFrEF) and heart failure with preserved ejection fraction (HFpEF). Their use is no longer limited to patients with concomitant diabetes (34). Increasingly, studies have shown that even in myocardial infarction patients who have not yet developed clinical heart failure, SGLT2 inhibitors may provide potential benefits (35–37).

SGLT2 inhibitors can improve cardiac structure and function through multiple pathways and mechanisms, despite the minimal expression of SGLT2 inhibitors in myocardial tissue. They optimize myocardial energy metabolism, enhancing the utilization of fatty acids and ketone bodies, while also improving endothelial function and vasodilation, thereby maintaining cardiac contractile function (38, 39). Additionally, SGLT2 inhibitors can regulate the activity of the sodium-hydrogen exchanger, inhibiting myocardial sodium load and calcium overload, thus delaying myocardial hypertrophy and contractile dysfunction (40). At the molecular level, SGLT2 inhibitors upregulate Sirtuin-1 and its downstream signaling pathways, promoting autophagic flux, reducing oxidative stress, and preserving mitochondrial structure and function integrity (41, 42). The synergistic effects of these multifaceted mechanisms not only enhance cardiac energy efficiency, help stabilize atherosclerotic plaque, improve endothelial function, and reduce the risk of acute coronary events, but also mitigate myocardial remodeling and the risk of arrhythmias (36, 43). Notably, SGLT2 inhibitors may further reduce the risk of developing heart failure by regulating non-osmotic sodium storage and directly acting on myocardial cells (44). This complex and interconnected mechanistic network provides direct evidence for the role of SGLT2 inhibitors in improving post-myocardial infarction ventricular remodeling, further supporting the biological rationale for their cardioprotective effects.

This study systematically assessed the efficacy and safety of adding sodium-glucose cotransporter 2 inhibitors (SGLT2i) to conventional therapy in patients with myocardial infarction. Analysis of 13 randomized controlled trials involving a total of 22,238 patients revealed that the combination of SGLT2i and conventional therapy significantly reduced the risk of the primary endpoint—heart failure-related hospitalizations—by 25% (RR = 0.76, 95% CI 0.68–0.84, *p* < 0.00001) and was also associated with a 13% reduction in the risk of major adverse cardiovascular events (RR = 0.87, 95% CI 0.79–0.96, *p* = 0.004). Notably, the addition of SGLT2i therapy was linked to a 24% reduction in the risk of renal impairment (RR = 0.77, 95% CI 0.66–0.89, *p* = 0.0006) and a 40% reduction in the risk of poor blood glucose control (RR = 0.60, 95% CI 0.43–0.85, *p* = 0.003), with no significant increase in the risk of urinary tract infections or liver function impairment.

Notably, the cardiovascular benefits observed in this study were not dependent on whether patients had concomitant diabetes, which aligns with previous research indicating that “the cardiovascular protective effects of SGLT2 inhibitors are independent of their glucose-lowering effects” (45). Although direct evidence in the elderly population remains relatively limited, existing studies generally support the potential benefits of SGLT2 inhibitors in patients across different age groups and risk levelsh (46). However, age was not stratified in this study. Subgroup analyses further confirmed that these benefits were, to some extent, independent of the specific type of SGLT2 inhibitor used, further highlighting the potential of this class of drugs in treating this population.

Some large randomized controlled trials, such as EMPACT-MI (16), did not observe significant statistical differences in their primary composite endpoints, which has led to discussions about the clinical value of SGLT2 inhibitors in patients with myocardial infarction. However, these studies often used “first heart failure hospitalization or cardiovascular death” as their primary endpoint, with relatively limited follow-up duration and a low overall event rate, which may have limited statistical power. Additionally, differences in baseline risk of the enrolled populations, timing of SGLT2 inhibitor initiation, and endpoint definitions across studies may also affect the comparability of results. In contrast, by synthesizing data from multiple randomized controlled trials, this study increased statistical power and showed a beneficial trend of SGLT2 inhibitors in reducing heart failure-related hospitalizations, improving cardiac function indices, and lowering the risk of adverse cardiovascular events, thus providing more robust evidence to support their potential clinical value in the myocardial infarction population.

## Analysis of limitations

This study also has several limitations that need to be addressed. (1) Although all included studies were randomized controlled trials, there were still differences across studies in the definition of myocardial infarction, patient risk characteristics, timing of SGLT2 inhibitor initiation, and follow-up duration, which may have influenced some of the outcome results. (2) This study included “studies not specifically designed for MI,” and only a subset of the population from these studies was analyzed. Despite performing sensitivity analyses, some level of bias may have been unavoidable, which could affect the strength of the evidence. (3) Only 3–4 studies reported data on poor blood glucose control, LDL-C, urinary tract infections, LVEF, or NT-proBNP, and the related results should be interpreted with caution. (4) This study primarily conducted a pooled analysis of the data from the included studies, lacking detailed individual-level patient information, which limited further refined analysis for specific subgroups (e.g., different age groups or patients with varying baseline left ventricular ejection fraction levels). (5) While the addition of SGLT2 inhibitors reduced the incidence of MACE in myocardial infarction patients, due to limited data, we could only conclude that SGLT2 inhibitors had no impact on cardiovascular death, without identifying the specific factors influencing MACE outcomes. (6) Some secondary outcomes had a limited number of studies included, and their results should still be interpreted with caution. Future prospective, large-scale randomized controlled trials are needed to clarify the optimal timing and long-term benefits of SGLT2 inhibitors in different stages of myocardial infarction and in various risk populations (7). In this study, based on previous research findings, we divided patients into two groups for stratified analysis: “SGLT2i treatment initiated within 8 weeks after MI” and “SGLT2i treatment initiated after 8 weeks post-MI,” as ventricular remodeling is most pronounced around 8 weeks following myocardial infarction. We assessed the impact of the timing of SGLT2i initiation on clinical outcomes. While this time point is supported by some literature, it may still be influenced by individual patient differences, treatment delays, and other factors. Its applicability requires further research and validation.

## Conclusion

This systematic review and meta-analysis integrated currently available evidence from randomized controlled trials and demonstrated that, among patients with myocardial infarction, the addition of SGLT2 inhibitors to conventional therapy was associated with a significant reduction in the risk of hospitalization for heart failure. This benefit was consistent across predefined clinical subgroups and was not materially influenced by the timing of myocardial infarction, the presence of type 2 diabetes mellitus, or the specific SGLT2 inhibitor used, with an overall favorable safety profile. In addition, SGLT2 inhibitor therapy was associated with improvements in cardiac function parameters and a reduced risk of major adverse cardiovascular events. Collectively, these findings support the potential cardiovascular protective role of SGLT2 inhibitors in patients with myocardial infarction and suggest that their therapeutic window may extend to the preclinical stage before overt heart failure develops.

## Data Availability

The original contributions presented in the study are included in the article/Supplementary Material, further inquiries can be directed to the corresponding authors.
