# Supplementary material for: Clinical effects of sodium–glucose cotransporter 2 inhibitors combined with conventional therapy in myocardial infarction: a systematic review and meta-analysis of randomized controlled trials
Source: Front Cardiovasc Med. 2026 Apr 21;13:1797628. doi: 10.3389/fcvm.2026.1797628 (PMC13138949; doi:10.3389/fcvm.2026.1797628)
Supplement: Supplementary file 1 [file Supplementaryfile1.docx]

**Search strategie：**

Pubmed:

((((((((((1-chloro-4-(glucopyranos-1-yl)-2-(4-(tetrahydrofuran-3-yloxy)benzyl)benzene[Title/Abstract]) OR (BI 10773[Title/Abstract])) OR (BI-10773[Title/Abstract])) OR (BI10773[Title/Abstract])) OR (Jardiance[Title/Abstract])) OR ("empagliflozin" [Supplementary Concept])) OR (((((((((2S,3R,4R,5S,6R)-2-(4-chloro-3-(4-ethoxybenzyl)phenyl)-6- (hydroxymethyl)tetrahydro-2H-pyran-3,4,5-triol[Title/Abstract]) OR (2-(3-(4-ethoxybenzyl)-4-chlorophenyl)-6-hydroxymethyltetrahydro-2H-pyran-3,4,5-triol[Title/Abstract])) OR (BMS 512148[Title/Abstract])) OR (BMS-512148[Title/Abstract])) OR (BMS512148[Title/Abstract])) OR (Farxiga[Title/Abstract])) OR (Forxiga[Title/Abstract])) OR ("dapagliflozin" [Supplementary Concept]))) OR (((((Invokana[Title/Abstract]) OR (Canagliflozin, Anhydrous[Title/Abstract])) OR (1-(Glucopyranosyl)-4-methyl-3-(5-(4-fluorophenyl)-2-thienylmethyl)benzene - T777973[Title/Abstract])) OR (Canagliflozin Hemihydrate[Title/Abstract])) OR ("Canagliflozin"[Mesh]))) OR (("ertugliflozin" [Supplementary Concept]) OR (((((5-(4-chloro-3-(4-ethoxybenzyl)phenyl)-1-hydroxymethyl-6,8-dioxabicyclo(3.2.1)octane-2,3,4-triol[Title/Abstract]) OR (PF 04971729[Title/Abstract])) OR (PF-04971729[Title/Abstract])) OR (PF04971729[Title/Abstract])) OR (Steglatro[Title/Abstract])))) OR (((2S,3R,4R,5S,6R)-2-(4-chloro-3-(4-(2-cyclopropoxyethoxy)benzyl)phenyl)-6-(hydroxymethyl)tetrahydro-2H-pyran-3,4,5-triol[Title/Abstract]) OR ("bexagliflozin" [Supplementary Concept]))) AND ((((((((((((((Infarction, Myocardial[Title/Abstract]) OR (Infarctions, Myocardial[Title/Abstract])) OR (Myocardial Infarctions[Title/Abstract])) OR (Heart Attack[Title/Abstract])) OR (Heart Attacks[Title/Abstract])) OR (Myocardial Infarct[Title/Abstract])) OR (Infarct, Myocardial[Title/Abstract])) OR (Infarcts, Myocardial[Title/Abstract])) OR (Myocardial Infarcts[Title/Abstract])) OR (Cardiovascular Stroke[Title/Abstract])) OR (Cardiovascular Strokes[Title/Abstract])) OR (Stroke, Cardiovascular[Title/Abstract])) OR (Strokes, Cardiovascular[Title/Abstract])) OR ("Myocardial Infarction"[Mesh]))
